# Mass spectrometry-based investigation of measles and mumps virus proteome

**DOI:** 10.1186/s12985-018-1073-9

**Published:** 2018-10-16

**Authors:** Dora Sviben, Dubravko Forcic, Beata Halassy, Günter Allmaier, Martina Marchetti-Deschmann, Marija Brgles

**Affiliations:** 1 Centre for Research and Knowledge Transfer in Biotechnology, University of Zagreb, Rockefellerova 10, HR-10 000 Zagreb, Croatia; 2 Centre of Excellence for Viral Immunology and Vaccines, CERVirVac, Zagreb, Croatia; 3 Institute of Chemical Technologies and Analytics, TU Wien, Getreidemarkt 9, AT-1060 Vienna, Austria

**Keywords:** Measles virus, Mumps virus, Extracellular vesicles, Host cell proteins, Mass spectrometry

## Abstract

**Background:**

Measles (MEV) and mumps virus (MUV) are enveloped, non-segmented, negative single stranded RNA viruses of the family *Paramyxoviridae*, and are the cause of measles and mumps, respectively, both preventable by vaccination. Aside from proteins coded by the viral genome, viruses are considered to contain host cell proteins (HCPs). The presence of extracellular vesicles (ECVs), which are often co-purified with viruses due to their similarity in size, density and composition, also contributes to HCPs detected in virus preparations, and this has often been neglected. The aim was to identify which virus-coded proteins are present in MEV and MUV virions, and to try to detect which HCPs, if any, are incorporated inside the virions or adsorbed on their outer surface, and which are more likely to be a contamination from co-purified ECVs.

**Methods:**

MUV, MEV and ECVs were purified by ultracentrifugation, hydrophobic interaction chromatography and immunoaffinity chromatography, proteins in the samples were resolved by SDS-PAGE and subjected to identification by MALDI-TOF/TOF-MS. A comparative analysis of HCPs present in all samples was carried out.

**Results:**

By proteomics approach, it was verified that almost all virus-coded proteins are present in MEV and MUV particles. Protein C in MEV which was until now considered to be non-structural viral protein, was found to be present inside the MeV virions. Results on the presence of HCPs in differently purified virus preparations imply that actin, annexins, cyclophilin A, moesin and integrin β1 are part of the virions.

**Conclusions:**

All HCPs detected in the viruses are present in ECVs as well, indicating their possible function in vesicle formation, or that most of them are only present in ECVs. Only five HCPs were constantly present in purified virus preparations, regardless of the purification method used, implying they are likely the integral part of the virions. The approach described here is helpful for further investigation of HCPs in other virus preparations.

**Electronic supplementary material:**

The online version of this article (10.1186/s12985-018-1073-9) contains supplementary material, which is available to authorized users.

## Background

Measles (MEV) and mumps (MUV) viruses are non-segmented, negative single stranded RNA viruses from the family *Paramyxoviridae* which cause measles and mumps, respectively. MEV and MUV virions are enveloped with lipid membrane derived from the host cell plasma membrane and are pleomorphic in shape, with diameter in range 100–900 nm [1–5].

Genomic RNAs of MEV and MUV have 15,894 and 15,384 base pairs coding in total for 8 and 9 viral proteins, respectively. Viral RNA is packed into a filamentous complex called nucleocapsid by the nucleoprotein (denoted NP for MUV and N for MEV) which interacts with large polymerase (L) through phosphoprotein (P). This core unit, also referred to as ribonucleocapsid, is linked to the matrix protein (M) found directly below virion’s lipid bilayer [6–9].

Lipid bilayer is spiked with two types of glycoproteins: attachment proteins, hemagglutinin – neuraminidase (HN) in MUV and hemagglutinin (H) in MEV, are responsible for virus attachment to the surface of the host cell, and fusion protein (F) is responsible for the fusion of virus and cell membrane in both viruses [10]. F protein of MEV and MUV is synthesized as an inactive precursor F_0._ Its active form consists of two fragments F_1_ and F_2_ linked with disulphide bridges formed after cleavage by host cell protease furin which specifically recognizes the RRHKR motif [2, 3].

In both MEV and MUV, the transcription of the P gene results in three mRNA transcripts coding for P/V/I proteins in MUV and P/V/C proteins in MEV [11, 12]. V/I and V/C are often considered as non-structural proteins, and it was reported that they are not necessary for virus replication in Vero cells [13–16].

MUV genome codes for another, small hydrophobic protein (SH). This non-structural protein also seems not to be necessary for MUV replication [17], and is considered to be a membrane protein present in the lipid bilayer [18].

MEV and MUV genomes are explored in detail, but the studies of their proteomes were mostly carried out during 1970s and 1980s, prior to development of high sensitivity “soft ionization” mass spectrometric methods [19–31]. In these early studies, up to 6 viral proteins were usually detected – H/HN, P, N/NP, M and F were readily confirmed in virus samples, and some groups also detected the L protein in their preparations [22, 26, 29–31]. These early investigations relied on gel electrophoresis for determination of molecular masses of proteins, combined with labelling newly synthesized proteins by radioactive amino acids (^14^C-labeled amino acid mixtures, ^3^H-leucine or ^35^S-methionine), and detection of glycoproteins by incorporation of ^3^H-glucosamine. Recently, MUV proteome was investigated by mass spectrometry for the first time and the presence of 6 virus polypeptides was confirmed: L, HN, NP, P, M and V [32].

Apart from virus-coded proteins, it is considered that various enveloped viruses incorporate also host cell proteins (HCPs), inside the viral particle and in the lipid bilayer [33]. Presence of HCPs in virus preparations is important both for basic research of the biology of these viruses, as well as for the vaccine production in industry because it means that the vaccine is not only carrying viral, but also HCP antigens [34]. Early investigations of MEV and MUV already reported the presence of cellular actin in virions [21, 26, 28], and additional HCPs such as fibronectin, clathrin and histones were detected in MUV samples investigated recently [32]. There are indications that some HCPs are specifically taken up by the viral particles, by direct interactions with viral proteins [34–36], but HCPs might also be incorporated non-specifically [37], can be adsorbed to the outer surface of the virion, or be present as contamination arising from the presence of cellular debris or extracellular vesicles (ECVs) in virus preparations [38, 39]. ECVs are produced by virtually all types of cells [40], and their similarity to viruses in size, density, and composition (e.g. proteins, lipids, nucleic acids), as well as the lack of clear line between ECVs and different types of non-infectious virus particles [41], makes their detection and thus separation from viruses extremely difficult. Some attempts to obtain ECV-free virus preparations have been made by treating the samples with proteases or by CD45 immunoaffinity depletion followed by density gradient ultracentrifugation [34, 37]. However, the presence of ECVs is still often disregarded in papers discussing virus proteomes, or they are mentioned as minor contaminants [42] which might not always be the case. There are results that imply that, if production of ECVs in non-infected and infected cells is the same, about one third of particles in virus suspensions are ECVs [43]. The main reasons for disregarding the presence of ECVs are probably problems with detecting and separating ECVs from viruses, combined with lack of awareness that both types of particles are secreted simultaneously. However, this raises questions about the conclusions drawn from such reports regarding HCPs present inside the virions.

## Methods

The aim of this research was to identify which virus coded proteins are present in MEV and MUV, and to detect which HCPs, if any, are attached to or potentially incorporated inside the virions. Since presence of ECVs in virus samples complicates this assessment, for the first time the evaluation of which HCPs might be part of the virions was carried out by comparison of HCPs detected in virus preparations purified by different purification methods (immunoaffinity chromatography, IAC, hydrophobic interaction chromatography, HIC, and ultracentrifugation, UC), and with HCPs present in ECVs produced by non-infected Vero cells. An attempt to evaluate which HCPs might be part of the virions, and which are more likely to be contaminants, was made under the hypothesis that HCPs present in all viral samples, regardless of the purification method used, are more likely to be incorporated in or attached to virions, whereas others are probably contaminants arising from ECVs co-purified with viruses. To support the hypothesis that HCPs which are detected only in some virus preparations are arising from the ECVs, the proteomes of ECVs purified from the supernatants of the non-infected Vero cells were also analysed and compared to proteomes of MEV and MUV.

### Cell cultures

Vero cell culture (African green monkey kidney cells) was obtained from European Collection of Animal Cell Culture (ECACC) and maintained in minimum essential medium with Hank’s salts (MEM-H) (AppliChem) supplemented with 10% (*v*/v) FCS (Invitrogen) and 50 μg neomycin mL^− 1^ (Gibco-Life Technologies).

### Virus production and purification

MEV strain Edmonston-Zagreb and MUV strain L-Zagreb were obtained from Institute of Immunology, Zagreb, Croatia. Vero cells were infected with MEV or MUV in suspension at m.o.i. of 0.005 or 0.001, respectively, in MEM-H with 2% (v/v) FCS. The medium was replaced with medium without FCS after 24 h, and virus was further grown until cytopathic effect was observed.

The culture supernatant was collected and clarified by microfiltration through 0.45 μm PVDF syringe filter (Millipore). Viruses were then subjected to purification by UC, HIC or IAC as previously described [5, 43, 44]. Briefly, viruses were purified by HIC on CIM OH monolithic column (column volume, CV 1 mL, channel size 6 μm) (BIA Separations) with binding buffer 50 mM HEPES, 1.0 M (NH_4_)_2_SO_4_, pH 7.3 and eluted by step gradient elution with 0.5 M and 0 M (NH_4_)_2_SO_4_ in 50 mM HEPES. In IAC, MUV suspension was loaded on CIM epoxy monolithic column coupled with polyclonal anti-MUV antibodies (CV 1 mL, channel size 6 μm) (BIA Separations) with binding buffer 20 mM MOPS, 0.15 M NaCl, pH 7.3 and eluted with solutions containing 0.75 M Arg/ 0.75 M imidazole or 0.75 M Arg/ 0.75 M Ser, all at pH 7.3. Obtained eluates were additionally concentrated by UC for 2 h at 141,000×*g* to remove free or loosely bound proteins from the surface of the particles, which might co-purify during chromatography. For UC purification, only one-step UC for 2 h at 141,000×*g* was used and the obtained pellets were resuspended in 250 μL PBS.

Supernatants obtained from non-infected Vero cell culture containing only ECVs were purified in the same way as described for virus suspensions.

### Virus quantification

Quantification of viable virus particles was performed using a CCID_50_ assay as described elsewhere [45, 46]. Total particle concentration and particle size in virus and ECV samples was determined by Nanoparticle Tracking Analysis (NTA), using a Nanosight LM10 instrument (Malvern) which consists of a conventional optical microscope, sCMOS camera and a LM10 unit with a red laser light source. High particle concentration samples were diluted with PBS prior to measurements to achieve concentration range 2–8 × 10^8^ particles/mL. For each sample three 60 s videos of particles under Brownian motion were obtained with camera level fixed at 10 and analysed with detection threshold 5 using NTA 3.2 software.

### Protein analysis

Virus samples were subjected to SDS gel electrophoresis under denaturing and reducing conditions in 1× NuPAGE LDS Sample Buffer (Invitrogen) with 10% NuPAGE Reducing Agent (Invitrogen). NuPAGE 4–12% Bis-Tris precast gels (Invitrogen) were used with MES running buffers and Novex Sharp Pre-stained Protein Standard (Invitrogen) in an XCell Sure Lock system (Invitrogen) according to manufacturer’s instructions. Protein band detection was performed by using acidic Coomassie Brilliant Blue R250 solution or by silver staining as described previously [47].

Protein bands were excised from the gel, digested by trypsin (porcine, Roche), peptides were extracted from the gel, purified and prepared for MS analysis as described previously [48].

### MALDI MS analysis

Measurements were performed on UltrafleXtreme (Bruker) mass spectrometer in positive, reflectron ion mode. The instrument is equipped with 2 kHz SmartBeam solid state laser (355 nm), and the applied acceleration voltage was 8 kV in the positive mode. MS/MS spectra were obtained in the LIFT mode with the isolation of the monoisotopic peak. Obtained spectra were processed using FlexAnalysis (3.4.76.0) and BioTools (3.2. SR3) and identification searches were performed against NCBIprot database “Other viruses” and “Primates” (release 217, 12/2016 and release 221, 08/2017 with 198,565,475 and 203,180,606 sequences, respectively), and against contaminants database. Following parameters were used during searches: precursor ion mass tolerance ±200 ppm, product ion mass ± 1.0 Da, two missed trypsin cleavages, constant carbamidomethylation of Cys and variable modifications: N-acetylation, C-amidation, ammonia loss from N-terminal Cys, modification of N-terminal Gln to pyro-Glu, oxidation of Met, His or Trp, phosphorylation of Ser, Thr or Tyr. Proteins were identified by peptide mass fingerprint (PMF) and peptide sequencing, with a minimum of 4 sequenced peptides identifying the protein.

## Results

The genomes of MEV and MUV are well explored, but the proteome of MUV was only recently investigated by mass spectrometry (MS) [32], which is currently the dominant method in the field [49]. This is the first investigation of MEV proteome by MS, to the best of author’s knowledge. MEV and MUV genomes encode for 8 and 9 proteins (Additional file 1, Tables S1 and S2, respectively), but it is important to investigate if all or just a subset of them are synthesized and present in the viral particle. It is also essential to try to identify which HCPs are a part of the virions, and which are present just as contaminations in virus preparations. It should be mentioned here that some of the viral proteins may also be included into ECVs inevitably present in the virus preparations [41].

MEV and MUV proteomes of viruses purified by UC and HIC, and for MUV also by IAC, were analysed and compared. A comparison of the proteomes of viruses purified by different purification methods, as well as the comparison with proteomes of ECVs purified from non-infected cell culture supernatants by the same methods, was carried out to try and determinate which HCPs are virion-associated. All viral samples contained infective particles (determined by CCID_50_ assay), and data regarding total and infective particles of all analysed samples is given in Table 1.

**Table 1:** Total and infective particle concentrations in the analysed samples

	UC			HIC^ab^					IAC^a^		
				E1			E2				
	*n*	Total particles/ log_10_ units mL^−1^	Infective particles/ lo_10_ CCID_50_ units mL^−1^	*n*	Total particles/ log_10_ units mL^−1^	Infective particles/ log_10_ CCID_50_ units mL^− 1^	Total particles/ log_10_ units mL^− 1^	Infective particles/ log_10_ CCID_50_ units mL^− 1^	*n*	Total particles/ log_10_ units mL^− 1^	Infective particles/ log_10_ CCID_50_ units mL^− 1^
MEV	3	(A) 11.934 (B) 11.387 (C) 11.873	9.135 9.765 7.705	3	8.619	5.614	9.113	6.333	0	NA	NA
MUV	3	(A) ND (B) 12.002 (C) 12.310	6.422 7.946 7.085	3	9.026	7.835	9.462	8.174	2	(A) ND (B) 9.200	(A) ND (B) 6.832
ECV	2	(B) 11.700 (C) 11.939	NA NA	1	8.379	NA	8.874	NA	1	8.798	NA

*ND* not deterined

*n* – number of samples analysed; (A), (B), (C) – data corresponding to separately prepared samples denoted in the same way in corresponding figures

^a^ - pooled sample from 4 subsequent days;

^b^ - calculated average value for the pool after concentration by UC (% recovery calculated based on UC data presented in [5]); only data for the representative sample in Figs. 3 and 4 are given here

### Samples purified by ultracentrifugation

#### MEV

**Fig. 1:**
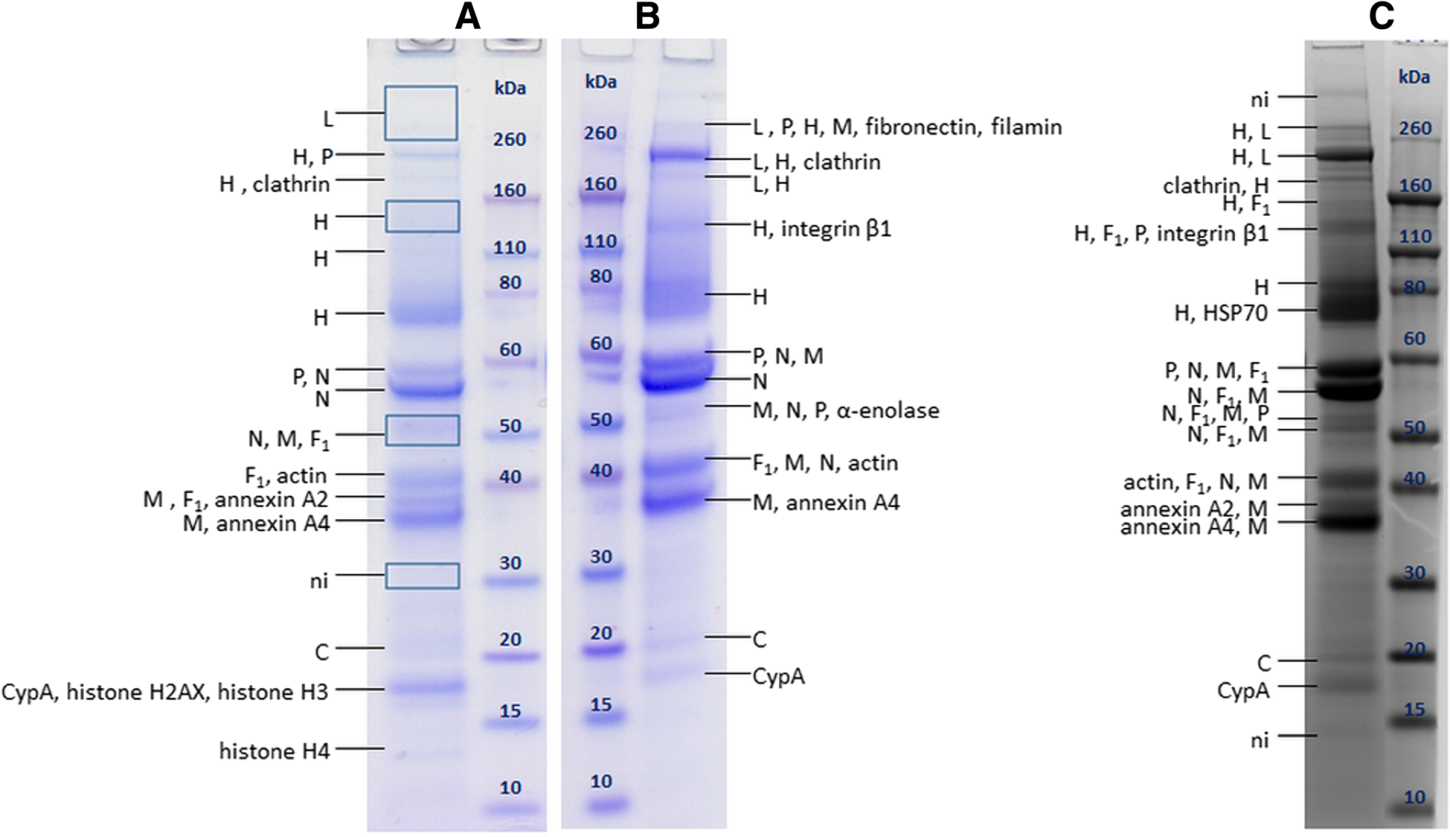
SDS-PAGE of MEV sample purified by UC with protein annotations after MALDI-TOF/TOF MS analysis. **a**, **b** and **c** represent three separately prepared samples for which the data are listed in Table 1. CypA – cyclophilin A, HSP – heat shock protein, ni – not identified. Rectangles denote areas where large gel pieces (possibly containing multiple but very faint bands) were excised

MALDI-TOF/TOF-MS analysis of MEV purified by UC in three separately prepared samples (Fig. 1) confirmed the presence of 7 out of 8 viral proteins: L, H, P, N, F, M and C. Protein C was detected for the first time in all three samples, at the apparent molecular mass (MM) which corresponds well to its theoretical molecular mass calculated from the amino acid sequence (MM_aa_) of 21 kDa.

Protein H was found in 5 to 7 bands, at MMs of 70 kDa and higher, which is higher than its MM_aa_ (69 kDa).

Protein N was found in 3 to 5 different bands at MMs equal or lower than MM_aa_ (59 kDa), indicating the presence of truncated forms. Analysis of peptide mass fingerprint (PMF) and MS/MS spectra revealed that, for the 56 kDa band, peptides from positions 33–521 were sequenced, indicating that this protein is not C-terminally truncated (Additional file 1: Figure S1), but could be N-terminally truncated. However, comparison of peptides detected in the PMF spectra of the rest of the bands does not provide further evidence to support or rebut this hypothesis, hence further investigation is needed.

Protein F appears in multiple bands at MMs equal and higher than its MM_aa_ (Fig. 1(a) and (c)). Peptides corresponding to fragment F_1_ were found in all bands, however successfully sequenced peptides did not give any further insights on protein forms present in different bands (Additional file 1: Figure S2). Multiple bands containing F_1_ are present in the 50–55 kDa range, however their origin is unclear for now.

M protein also appears in multiple bands, at MMs equal and higher than MM_aa_ (37 kDa). Here, several bands are consistently present in the 40–60 kDa range.

P protein is found in 2 to 3 bands, at approximately 53 kDa corresponding well to MM_aa_ of P (54 kDa), at approximately 60 kDa, and in bands at higher MMs (110 kDa and more).

#### MUV

**Fig. 2:**
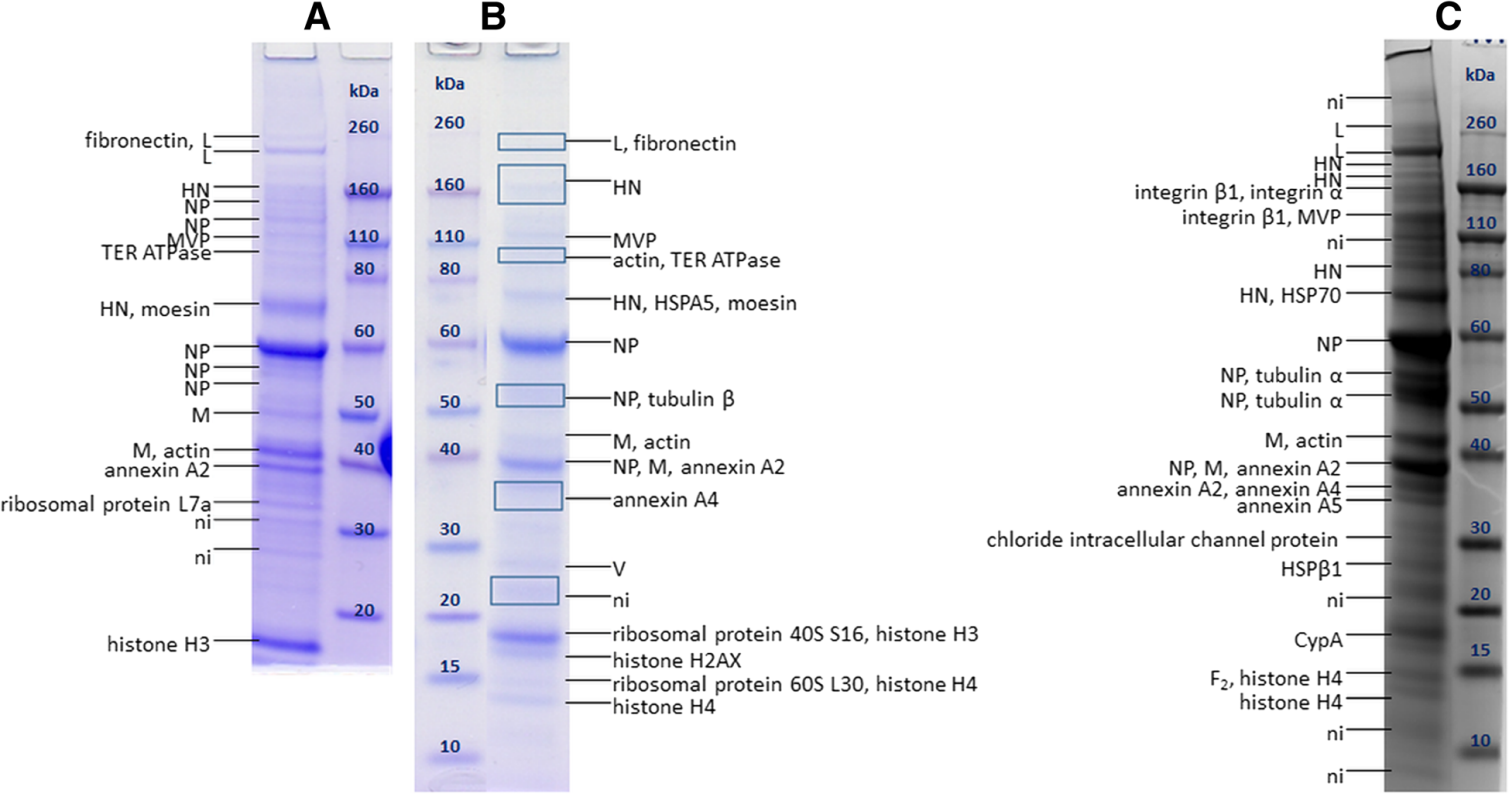
SDS-PAGE of MUV sample purified by UC with protein annotations after MALDI-TOF/TOF MS analysis. **a**, **b** and **c** represent three separately prepared samples for which the data are listed in Table 1. MVP – major vault protein, TER ATPase – transitional endoplasmatic reticulum ATPase, HSP – heat shock protein, CypA – cyclophilin A, ni – not identified. Rectangles denote areas where large gel pieces (possibly containing multiple but very faint bands) were excised

MUV purified by UC (Fig. 2) contained numerous bands. Here, 6 viral proteins were detected: L, HN, NP, M, V and F_2_.

Protein HN was detected at MMs ranging from 70 to 200 kDa, in 2 to 4 bands, which are higher than its MM_aa_ (64 kDa).

Protein NP was detected in 3 to 4 bands at MMs equal or lower than MM_aa_, ranging from approximately 39 to 61 kDa. Comparison of detected peptides in the PMF spectra (Additional file 1: Figure S3) might indicate C-truncation of proteins present in the bands at MMs lower than 61 kDa.

F_0_ precursor and F_1_ fragment remained undetected in UC purified MUV, but F_2_ fragment was for the first time successfully detected in the sample in Fig. 2(c) at MM slightly higher than its MM_aa_ (11 kDa). Proteins L and V was also detected in MUV, in the area corresponding to their MM_aa_.

### Samples purified by hydrophobic interaction chromatography

#### MEV

**Fig. 3:**
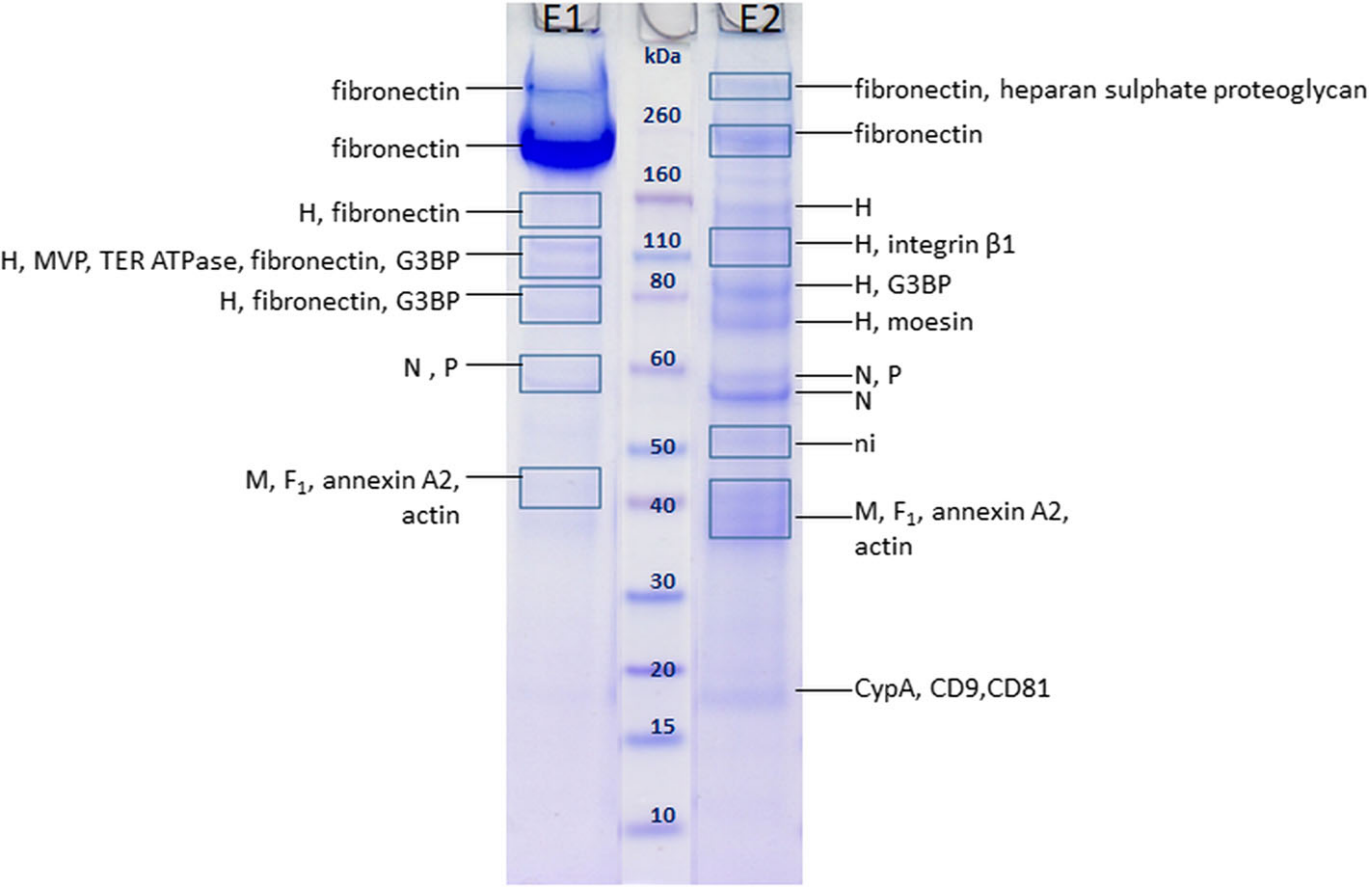
SDS-PAGE of a representative MEV sample purified by HIC with protein annotations after MALDI-TOF/TOF MS analysis. Three separate samples were analysed in total. E1 – eluate with 0.5 M (NH_4_)_2_SO_4_, 50 mM HEPES, E2 – eluate with 50 mM HEPES, MVP – major vault protein, G3BP – galectin-3-binding protein, CypA – cyclophilin A, TER ATPase – transitional endoplasmatic reticulum ATPase, ni – not identified. Rectangles denote areas where large gel pieces (possibly containing multiple but very faint bands) were excised

Analysis of MEV purified by HIC (Fig. 3) confirmed the presence of 5 out of 8 viral proteins: H, P, N, F and M. Viral protein H was detected in 3 and 4 bands in eluates E1 and E2, respectively, at MMs ranging from 70 to 170 kDa, which are higher than its MM_aa_ (69 kDa).

Protein N was detected in 2 bands - at approximately 59 kDa, corresponding well to MM_aa_ of N, and at 55 kDa.

Peptides corresponding to F_1_ fragment were again detected at MMs comparable to its MM_aa_ (47 kDa), whereas no protein bands were observed in the range where F_2_ should be present.

#### MUV

**Fig. 4:**
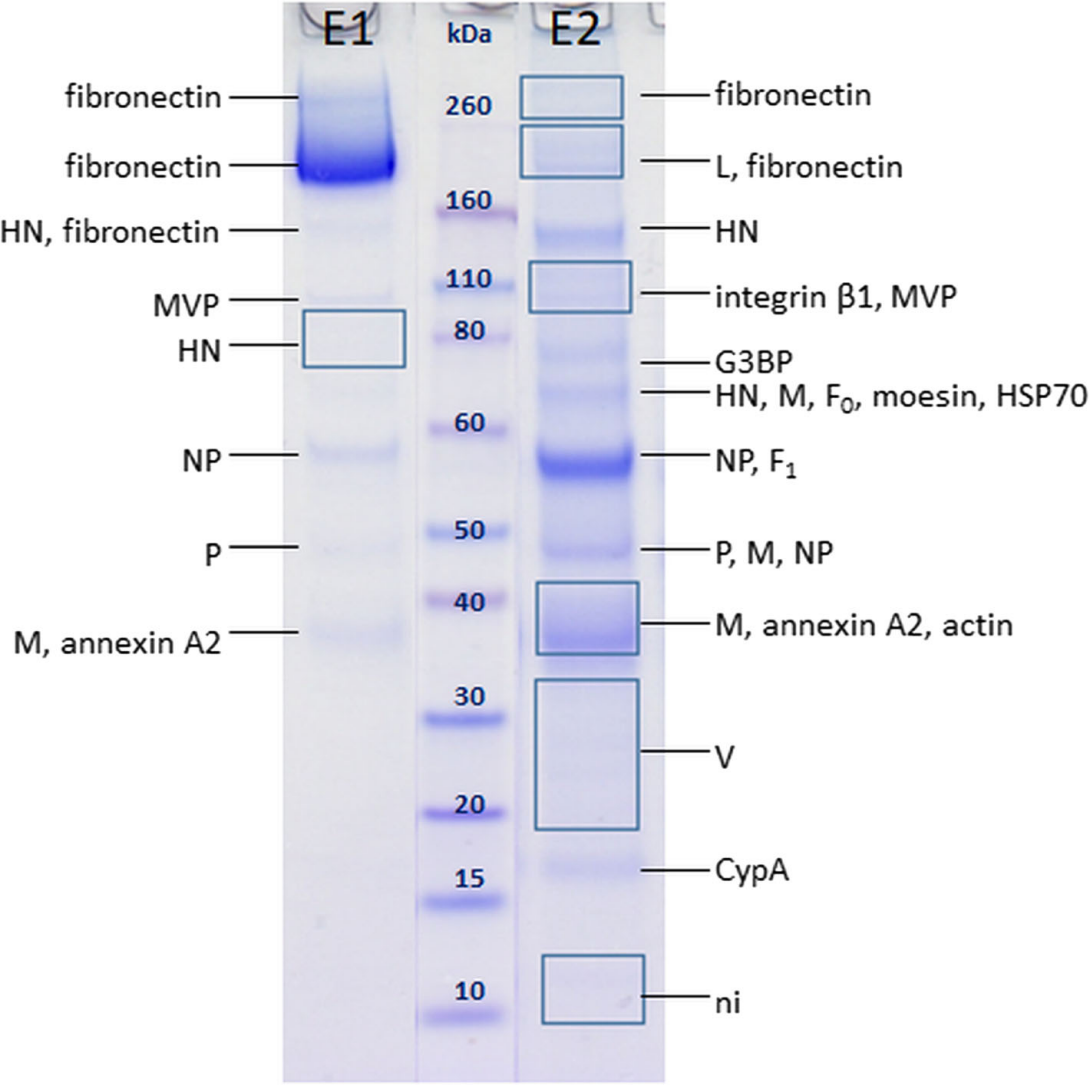
SDS-PAGE of a representative MUV sample purified by HIC with protein annotations after MALDI-TOF/TOF MS analysis. Three separate samples were analysed in total. E1 – eluate with 0.5 M (NH_4_)_2_SO_4_, 50 mM HEPES, E2 – eluate with 50 mM HEPES, MVP – major vault protein, G3BP – galectin-3-binding protein, CypA – cyclophilin A, ni – not identified. Rectangles denote areas where large gel pieces (possibly containing multiple but very faint bands) were excised

Analysis of MUV purified by HIC (Fig. 4) revealed for the first time the presence of more than 6 viral proteins in one sample. Here, 7 out of 9 viral proteins were successfully detected: L, HN, NP, P, M, F and V.

Protein HN (MM_aa_ 64 kDa) was detected in two bands at approximately 70 and 150 kDa.

Protein NP (MM_aa_ 61 kDa) was detected in two bands (approximately 46 and 56 kDa). Comparison of peptides present in the PMF spectra (Additional file 1: Figure S5) of these two bands indicates that both bands contain C-terminally truncated forms of NP. Peptides corresponding to the sequence positions 461–513 of 56 kDa protein were not sequenced, but only observed in the PMF spectra, however MM of protein truncated at position 513 would be 57 kDa which corresponds well to MM of the protein calculated from the position on the gel. When the same analysis was performed for protein truncated at position 400, the calculated MM again corresponds to the protein MM estimated from the gel (46 kDa). This hypothesis should however be further corroborated. Full-length NP appears not to be detected in this sample.

Protein F was detected in two bands around 57 kDa (only peptides of F_1_ fragment) and around 65 kDa (peptides from both F_1_ and F_2_ fragments).

### MUV purified by immunoaffinity chromatography

**Fig. 5:**
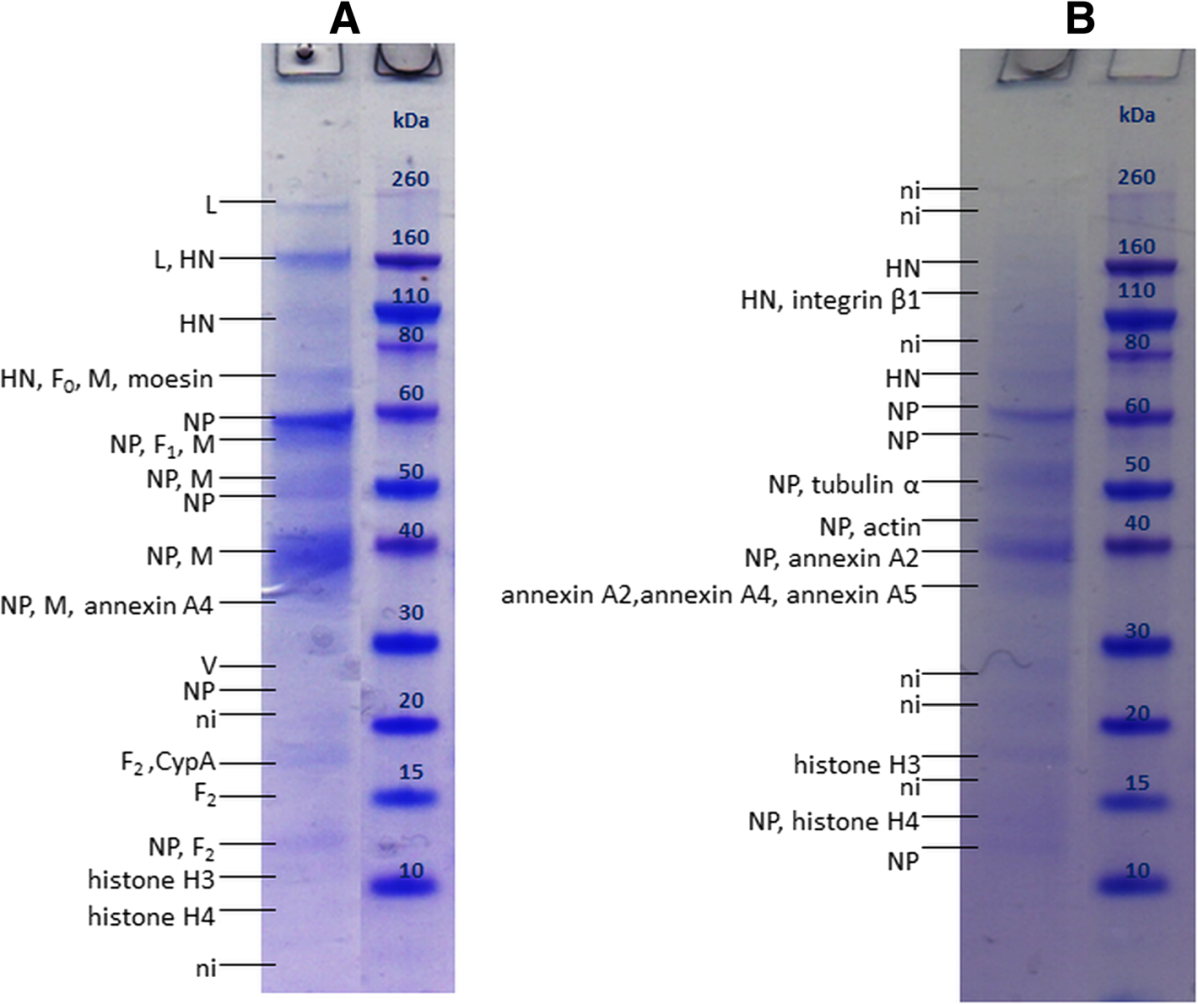
SDS-PAGE of MUV sample purified by IAC with protein annotations after MALDI-TOF/TOF MS analysis. **a** and **b** represent two separately prepared samples for which the data are listed in Table 1. CypA – cyclophilin A, ni – not identified

In MUV purified by IAC, 6 out of 9 viral proteins were detected: L, HN, F, M, NP and V (Fig. 5). For the first time all three forms of F protein in one sample were detected – F_0_, F_1_ and F_2_. F_2_ appears in 3 bands with quite different MMs, the lowest corresponding well to MM_aa_ (11 kDa).

Proteins HN and NP appear in multiple bands, with the same patterns observed in samples purified by UC and HIC. P protein was surprisingly again not detected. In the sample shown in Fig. 5(a), protein M is also present in multiple bands at higher MMs than its MM_aa_, similar as observed for MEV M in the UC purified sample. The underlying cause remains unknown.

### Comparison of protein composition of viruses and ECVs purified by different purification methods

**Fig. 6:**
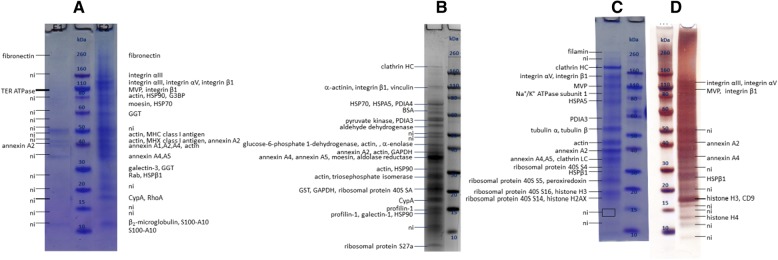
SDS-PAGE of ECVs purified by HIC **a**, UC **b**, **c**, and IAC (**d**) with protein annotations after MALDI-TOF/TOF MS analysis. MVP – major vault protein, G3BP – galectin-3-binding protein, CypA – cyclophilin A, TER ATPase – transitional endoplasmatic reticulum ATPase, HSP – heat shock protein, GGT – gamma-glutamyl transferase, PDI – protein disulphide-isomerase, BSA – bovine serum albumin, GAPDH – glyceraldehyde-3-phosphate dehydrogenase, GST – glutathione S-transferase, CypA – cyclophilin A, ni – not identified

Figure 6 shows ECVs purified by HIC (Fig. 6(a)), UC (Fig. 6(b) and (c)) and IAC (Fig. 6(d)). A concise list of HCPs found in all analysed MUV, MEV and ECV samples is given in Table 2. Some HCPs were detected in MUV and MEV only when UC purified samples were additionally analysed by 2D gel electrophoresis (Additional file 1: Figure S6 and Table S3).

**Table 2:** Comparative analysis of HCPs present in viruses and ECVs

Protein	MM^a^/kDa	UC			HIC						IAC	
					E1			E2				
		ECV	MUV	MEV	ECV	MUV	MEV	ECV	MUV	MEV	ECV	MUV
heparan sulphate proteoglycan	469									+		
filamin	280	+		+								
fibronectin	262	+	+	+	+	+	+	+	+	+		
clathrin heavy chain (HC)	192	+		+								
vinculin	124	+										
integrin α III	117		2D					+			+	
integrin α V	116	+	+					+			+	
Na^+^/K^+^ ATPase subunit 1	113	+										
α-actinin	103	+			+							
major vault protein (MVP)	99	+	+			+		+	+		+	
transitional endoplasmatic reticulum ATPase (TER ATPase)	89		+		+							
integrin β1	88	+	+	+				+	+	+	+	+
heat shock protein 90 (HSP90)	83	+			+			+				
protein disulphide-isomerase A4 (PDIA4)	73	+										
heat shock protein A5 (HSPA5)	72	+	+									
heat shock protein 70 (HSP70)	71	+	+	+				+	+			
moesin	68	+	+	2D				+	+	+		+
BSA (precursor)	66	+										
galectin-3-binding protein (G3BP)	65							+	+	+		
pyruvate kinase	62	+										
gamma-glutamyltransferase (GGT)	61							+				
glucose-6-phosphate 1-dehydrogenase	59	+										
protein disulphide-isomerase A3 (PDIA3)	57	+	2D									
aldehyde dehydrogenase	55	+										
tubulin β	50	+	+									
tubulin α	50	+	+	2D								+
α-enolase	47	+	2D	+								
actin	42	+	+	+			+	+	+	+		+
MHC class I antigen	41							+				
annexins A1,A2,A4,A5	39	+	+	+	+	+	+	+	+	+	+	+
aldolase reductase	36	+	2D									
glyceraldehyde 3-phosphate dehydrogenase (GAPDH)	36	+										
ribosomal protein 40S SA	33	+										
triosephosphate isomerase	31	+	2D	2D								
ribosomal protein L7a	30		+									
ribosomal protein 40S S4	30	+										
clathrin light chain (LC)	27	+										
chloride intracellular channel protein	27		+									
galectin-3	26		2D					+				
CD9, CD81	25									+		
ras-related protein Rab 5	24							+				
glutathione *S*-transferase (GST)	23	+	2D									
ribosomal protein 40S S5	23	+										
heat shock protein β1 (HSPβ1)	23	+	+	2D				+			+	
RhoA	22							+				
peroxiredoxin	22	+	2D	2D								
cyclophilin A (CypA)	18	+	+	+				+	+	+		+
ubiquitin-ribosomal protein S27a	18	+		2D								
ribosomal protein 40S S16	16	+	+									
ribosomal protein 40S S14	16	+										
galectin-1	15	+		2D								
profilin-1	15	+										
histone H3	15	+	+	+							+	+
histone H2AX	15	+	+	+								
β_2_-microglobulin	14	+										
ribosomal protein 60S L30	13		+									
S100-A11	12		2D					+				
histone H4	11		+	+							+	+
S100-A10	11							+				

^a^molecular mass of a monomer is given

2D – indicates that the protein was detected in 2D gel electrophoresis of UC purified MEV (Table 1, sample C and Fig. 1(C)) or MUV (Table 1, sample (C) and Fig. 2(C)), but not when these samples were resolved only by SDS-PAGE (for further information see Additional file 1: Figure S6 & Table S1)

Actin, annexins, cyclophilin A (CypA), integrin β1 and moesin were consistently found in both virus and ECV samples, regardless of the purification method. Fibronectin was found in MUV and MEV, as well as ECVs, and in fraction E1 of HIC purified samples it was present in very high concentrations.

## Discussion

### MEV

Protein C was detected in UC purified MEV. Until now it was only reported to be synthesized in Vero cells, but this was the first time it was found in MEV virions [29]. The intensities of the bands containing protein C indicate its low abundance in virus samples, which might explain why C was not detected in HIC purified MEV samples. V and C were until now considered non-structural MEV proteins, and were found not to be necessary for replication of MEV in Vero cells [2, 13, 14]. Therefore, it is very interesting that C is in fact packed into MEV virions. Protein V was not detected in MEV samples, possibly due to even lower abundance than C, or absence from the virions.

Detection of protein H in up to 7 bands in MEV samples implies the presence of various glycoforms as well as the presence of both monomer and dimer on the gel, as previously described [27].

Occurrence of protein N in multiple bands at MM equal and lower than MM_aa_ is in agreement with previous results which hypothesized that minor bands ranging from 40 to 55 kDa belong to breakdown products of N protein or its truncated forms [23, 32]. It is also interesting to notice that the intensity of the 55–56 kDa band in the samples is typically higher than of that at 59 kDa. This might be the result of changes in transcription or translation, or 59 kDa protein degradation by proteases during purification procedures [23], resulting in a more abundant 55–56 kDa form present on the gel. It is also interesting to notice that the analysis of peptides present in the PMF spectra of MEV purified by HIC did not indicate truncation of protein with apparent MM of 55 kDa (Additional file 1: Figure S4), however such result needs to be further corroborated.

Mature protein F consists of disulphide linked F_1_ (MM_aa_ 47 kDa) and F_2_ (MM_aa_ 13 kDa) fragments generated by F_0_ cleavage [2]. The uniqueness of MEV F in comparison to F protein of other paramyxoviruses is that all glycosylation sites appear to be on the F_2_ fragment [24–26]. It was proposed that F_2_ is usually not detected by Coomassie staining because of its diffuse nature due to its carbohydrate content [26]. F was found in multiple bands in UC purified mEV, at MMs equal and higher than MM_aa_. Bands containing F which were found around 40 kDa probably contain the non-glycosylated F_1_ fragment. Since MM_aa_ of non-glycosylated precursor F_0_ would be around 59 kDa, bands found around to 59 kDa may contain F_0,_ as previously reported [25, 29], however since no peptides corresponding to F_2_ fragment were detected, this cannot be confirmed. Multiple bands containing F_1_ were found in the 50–55 kDa range, however their origin is unclear for now. It is possible that some of them represent palmitoylated F_1_ [50] or degradation products of F_0_. Cross-contamination between bands as a cause of this phenomenon was excluded due to meticulous work in this and all other samples in which this occurs.

Protein M also appeared at several bands at MM equal and higher than its MM_aa_ (40–60 kDa). The doublet of bands around 37–39 kDa was previously reported for MEV M, and some smearing of M was reported even under reducing conditions [51]. Although biologically active form of M seems to be a dimer [51, 52], this does not explain the occurrence of these bands. The origin of M in multiple bands remains to be elucidated.

Protein P was detected at 2 to 3 bands in different MEV samples. The bands detected at approximately 53 kDa correspond well to MM_aa_ of P (54 kDa), thus likely corresponding to the protein without any posttranslational modifications. In the virions P is heavily phosphorylated [53], thus carrying large negative charge, therefore its migration in the gel should be retarded. This implies that the bands detected at approximately 60 kDa probably present posttranslationally modified P. P which was detected in bands at higher MMs (110 kDa and more) likely represents oligomers of P, since P is known to be a self-associated oligomer [54]. In previous reports P was also readily found in bands ranging from 65 to 70 kDa [23, 25, 29].

Viral protein L (MM_aa_ 248 kDa) was found at its corresponding MM in UC purified MEV, but in HIC purified MEV it remained undetected. This was probably due to its low abundance combined with its co-migration with much more abundant fibronectin, which might result in peptide desorption/ionization suppression.

### MUV

Results obtained for MUV in this study are similar to those previously published [32]. Although protein V is still often considered to be a non-structural protein and is not necessary for MUV replication [12, 16], it was shown to be present in all the samples analysed in this study, which is in line with previously published studies [32, 55].

Protein HN was detected in 2 to 4 bands at MMs ranging from 70 to 200 kDa, which is higher than its MM_aa_ (64 kDa). This indicates its presence as glycosylated monomer and dimer, as previously reported [32, 56]. The novelty is that in the sample on Fig. 2(c) HN was present in 4 bands, indicating that different glycoforms are likely present.

Protein NP was detected in up to 4 bands, at MM equal or lower than its MM_aa_, and the comparison of detected peptides in the PMF spectra of UC and HIC purified samples (Additional file 1: Figures S3 and S5, respectively) again indicates C-truncation of proteins present in the bands at MMs lower than 61 kDa, as described in our previous study [32]. Interestingly, in the present study, in HIC purified MUV full-length NP was not detected. When this is considered in parallel with the finding that lower MM forms of N are also more abundant in all MEV samples, it indicates some processes occur, either during virus production in the cells, or during virus purification, which result in more abundant truncated forms.

In this study, for the first time in UC and IAC purified MUV, fragment F_2_ of protein F was found at MM higher than it MM_aa_, indicating it is present in its glycosylated form. In HIC purified MUV, the band found around 57 kDa contained only peptides of F_1_ fragment, indicating it contains glycosylated F_1_, since its MM_aa_ is 47 kDa. The band around 65 kDa contains peptides from both F_1_ and F_2_ fragments, indicating the presence of glycosylated F_0_ precursor, since its MM_aa_ is 59 kDa [21, 22, 30].

In this study, in UC purified MUV samples protein P was not detected, which is unexpected since it was previously detected in MUV purified by UC [32]. Reports exist showing it is susceptible to protease degradation, which might explain its absence from the gel [23, 54].

In this study, the comparison of two HIC elution fractions, E1 and E2, reveals different protein patterns for both MEV and MUV, which are consistent with other findings such as total and infective particle number as seen in Table 1. It becomes clear that more viral proteins are present in E2 fraction which also contains more infective particles, as previously reported [43]. All these findings indicate that particles in fractions E1 and E2 differ significantly, possibly presenting different virus subpopulations [57].

### Host cell proteins in virus and ECV preparations purified by different purification methods

The presence of ECVs in virus preparations, which was often neglected, complicates determination of HCPs present only in virions and not in ECVs. Here, for the first time, this problem was addressed by comparative analysis of the results obtained for viruses and ECVs purified by different purification methods. Chromatographic techniques such as HIC and IAC result in virus preparations of higher purity in comparison to UC. This is easily observed through total-to-infective particle ratio in such samples, as well as in HCP content when compared to starting material [5, 47, 48]. Comparison of results obtained by different purification methods helps in estimating purification efficiency of these methods, as well as estimation if a method results in enrichment of certain particles (e.g. infective or non-infective virus particles, ECVs) or HCPs in comparison to other available methods.

ECVs were long ago shown to be a major contaminant of virus preparations, as well as a source of HCPs present in such virus preparations [38, 39]. ECVs are similar to MUV and MEV in size – ECVs produced by non-infected Vero cells used in our experiments have a mean diameter of 199 ± 3.8 nm (*n* = 39, updated data from [43]), whereas MUV and MEV have mean diameters of 215 ± 1.9 nm and 206 ± 2.5 nm, respectively (*n* = 67 and *n* = 68, respectively, updated data from [5]). Similarity of ECVs to enveloped viruses in size, density and composition makes the preparation of ECV-free virus samples virtually impossible by methods currently available, and in case that production of ECVs is not greatly affected during infection (increased or diminished), up to one third of particles in virus suspensions may be ECVs [43].

Here the evaluation of which HCPs might be part of the virions was carried out for the first time by comparing HCPs detected in virus preparations purified by different purification methods, and with HCPs present in ECVs produced by non-infected Vero cells. The hypothesis beneath this comparative analysis is that, if an HCP is virion-associated, it will be present in all virus samples, regardless of the purification method used. Otherwise, if the HCP is present in virus preparation obtained only by some purification methods, it is more likely to be a contamination arising from the ECVs present in the virus preparation. To confirm the incorporation of such HCPs into ECVs, a comparison to the proteome of purified ECVs from non-infected Vero cells was carried out under the hypothesis that the composition of ECVs produced by infected and non-infected Vero cells is the same. Although the protein composition of ECVs might change during infection, since ECVs produced by infected cells cannot be distinguished and separated from viral particles in the supernatant of the infected cell culture, the results presented here still give a valuable insight into which HCPs are more likely to be present in viral preparations due to their association with viral particles, and which due to inevitable presence of ECVs in virus preparations.

All of the proteins detected in ECV samples are considered to be exosome markers [58], except BSA which is likely a contaminant originating from FCS used in cell culture media during production of ECVs [59].

Fibronectin was found in MUV and MEV, as well as ECVs, and in fraction E1 of HIC purified samples it is present in very high concentrations. Since it is unlikely that any particles (virions or ECVs) would contain such high concentrations of fibronectin as seen in E1, fibronectin is probably co-purified from culture supernatant by HIC under used conditions, with most protein eluting in E1 fraction. Since samples were concentrated by UC prior to SDS-PAGE, free proteins present in the eluates should be removed as the forces during UC are not strong enough to pellet free proteins. However, high salt concentration used in HIC (in this case 1 M (NH_4_)_2_SO_4_) can cause fibronectin aggregation and even precipitation [60, 61]. Therefore, it is presumed that fibronectin has possibly formed large aggregates during HIC purification, which pelleted at 141,000×*g* used for UC. Fibronectin has previously been reported in MUV samples purified by UC [32], and it was also detected here in UC purified samples. Its presence in most samples might imply its involvement in particle formation; however, its absence from IAC purified MUV indicates that it might just be a contamination.

Actin, annexins (A1, A2, A4, A5) and cyclophilin A (CypA) are readily found in all samples which, combined with previous reports, strongly supports hypothesis that these proteins are in fact part of the virions. Presence of actin in MEV and MUV was previously reported [20, 21, 26, 31, 62]. It was shown that viruses use cytoskeletal proteins such as actin for transport of viral components inside the cell, as well as in virus budding and maturation [34, 62]. Actin was found to interact with ribonucleocapsid in MEV, and it also seems to interact with ribonucleocapsid, M and glycoproteins of some other paramyxoviruses [62–65]. It is likely responsible for maintaining the architecture of virions [42] and ECVs, hence its presence inside the particles is expected. In virions, it might have an additional function, e.g. it was found to be involved in genome transcription in several paramyxoviruses [64, 66].

Annexins are present in the cytoplasm but can also be bound to the plasma membrane surface. Annexin A2 binds cellular actin and is involved in its organization in the proximity of plasma membrane [34]. It is hypothesized that annexins as a part of viral particles aid the attachment of viruses on the host cells and fusion of virus and plasma membrane so it would be logical that they play the same role in the fusion of ECVs and cells. Although contradictory findings about the role of annexin A2 in virus infection have been published [67–70], it is possible that it might be important for infective virus formation in some cell lines [70].

Cyclophilin A (CypA) is highly abundant cytosolic protein acting as peptidyl-prolyl isomerase and is therefore often classified as a chaperone. Its presence in the particles might simply arise from its high abundance in the cytoplasm, but it could be incorporated into virions through interaction with viral proteins due to its chaperone function. It has been hypothesized that in some viruses it helps the formation of viral particles or uncoating after the infection, and it was also shown to be necessary for infective HIV-1 production [33, 35].

Integrin β1 and moesin were consistently found in both virus and ECV samples, regardless of the purification method. Their presence in IAC purified MUV supports the hypothesis that they are in fact included in virus particles. Integrin β1 was previously reported in vesicular stomatitis virus [71], whereas moesin was found in HIV [72]. Since integrins act as membrane receptors, and are involved in connecting extracellular matrix to cytoskeleton, and moesin is involved in the interaction of actin cytoskeleton with the plasma membrane, they are likely present at the virus budding sites and hence included into virions. Whether they have a specific role in the virus lifecycle itself remains unclear, although they seem to be important for the virus uptake to the cells [73, 74].

Virtually all of the detected HCPs have been previously reported as proteins present inside of purified virus particles [33–37, 42, 59, 62, 63, 65, 66, 71, 75–77]. However, one should be aware that co-purified ECVs contribute to detected HCPs [38, 39, 41]. Also, protein composition of ECVs might change during virus infection hence yielding ECVs of different composition, and this further underlines that co-purification of ECVs with viruses should not be neglected.

## Conclusions

Our research on MUV and MEV proteome resulted in detection of all viral proteins except V in MEV and SH and I in MUV, which might indicate that they are not incorporated in virions, especially since previous studies showed they were not necessary for virus replication. Different purification methods resulted in samples of different purity, but differences are also obvious between samples purified by the same methods. This is in line with already observed day-to-day variations [43]. Large-scale preparations would be needed to evaluate possible day-to-day proteome differences. Interestingly, many viral proteins occur in several bands, where only NP and N occur at theoretical molecular mass and lower, whereas all other multiple-band proteins occur at theoretical molecular mass and higher. All HCPs detected in the viruses are present in ECVs as well, indicating that they have some function in vesicle exit from the cell, or that they might only be present in ECVs. Presence of actin, annexins, CypA, integrin β1 and moesin in all virus samples indicates they are likely virion-associated, i.e. part of the virions, whereas further investigation is needed to confirm the incorporation of other HCPs into the virions.

## Additional file

Additional file 1: **Table S1.** Proteins coded by MEV genome (Edmonston-Zagreb strain, GenBank: AY486083.1). **Table S2.** Proteins coded by MUV genome (L-Zagreb strain, GenBank: AY685921.1). **Figure S1**. Sequence coverage of MEV N bands from UC purified MEV (Fig. 1(c)). **Figure S2.** Sequence coverage of MEV F bands from UC purified MEV (Fig. 1(c)). **Figure S3.** Sequence coverage of MUV NP bands from UC purified MUV (Fig. 2)(c)). **Figure S4.** Sequence coverage of MEV N bands from E2 fraction of HIC purified MEV (Fig. 3). **Figure S5.** Sequence coverage of MUV NP bands from fraction E2 of HIC purified MUV (Fig. 4). **Figure S6.** 2D gel electrophoresis of (a) MUV and (b) MEV purified by UC. **Table S3.** List of proteins detected in MEV and MUV samples purified by UC and analysed by 2D gel electrophoresis. (PDF 805 kb)

## Abbreviations

CypA: Cyclophilin A
ECV: Extracellular vesicle
F: Fusion protein
G3BP: Galectin-3 binding protein
H: Hemagglutinin
HCP: Host cell protein
HIC: Hydrophobic interaction chromatography
HN: Hemagglutinin-neuraminidase
IAC: Immunoaffinity chromatography
L: Large protein
M: Matrix protein
MALDI: Matrix-assisted laser desorption/ionization
MEV: Measles virus
MM: Molecular mass
MS: Mass spectrometry
MUV: Mumps virus
N/NP: Nucleoprotein
NTA: Nanoparticle tracking analysis
P: Phosphoprotein
SH: Small hydrophobic protein
UC: Ultracentrifugation
