# Supplementary material for: Mass spectrometry-based investigation of measles and mumps virus proteome
Source: Virol J. 2018 Oct 16;15:160. doi: 10.1186/s12985-018-1073-9 (PMC6192076; doi:10.1186/s12985-018-1073-9)
Supplement: Supplementary file 1 — Table S1. Proteins coded by MEV genome (Edmonston-Zagreb strain, GenBank: AY486083.1). Table S2. Proteins coded by MUV genome (L-Zagreb strain, GenBank: AY685921.1). Figure S1. Sequence coverage of MEV N bands from UC purified MEV (Fig. 1(c)). Figure S2. Sequence coverage of MEV F bands from UC purified MEV (Fig. 1(c)). Figure S3. Sequence coverage of MUV NP bands from UC purified MUV (Fig. 2)(c)). Figure S4. Sequence coverage of MEV N bands from E2 fraction of HIC purified MEV (Fig. 3). Figure S5. Sequence coverage of MUV NP bands from fraction E2 of HIC purified MUV (Fig. 4). Figure S6. 2D gel electrophoresis of (a) MUV and (b) MEV purified by UC. Table S3. List of proteins detected in MEV and MUV samples purified by UC and analysed by 2D gel electrophoresis. (PDF 805 kb) [file 12985_2018_1073_MOESM1_ESM.pdf]

Table S1. Proteins coded by MEV genome (Edmonston-Zagreb strain, GenBank: AY486083.1)

protein	CDS	#aa	M_r	pI	GI (NCBI)	UniProt entry
nucleoprotein, N	108 – 1685	525	58131	5.15	39932887	Q89933
phosphoprotein, P	1807 – 3330	507	53957	5.07	39932888	Q77M25
V	1807 – 2705	299	32074	4.80	39932889	Q77M24
C	1829 – 2389	186	21037	10.31	39932890	Q77M23
matrix protein, M	3438 – 4445	335	37749	9.24	39932891	Q6RXY8
fusion protein, F_0*	5449 – 7110	553	59864	8.87	39932892	O93055
fusion protein fragment 1, $F_1^{\#}$	-	438	47069	6.28	-	-
fusion protein fragment 2, $F_2^{\#}$	-	115	12812	11.46	-	-
hemagglutinin, H*	7271 – 9124	617	69162	6.74	39932893	Q9IC33
large polymerase, L	9234 – 15785	2183	247747	8.40	39932894	Q77M22

CDS - coding sequence; aa – amino acid; * - glycoproteins, real M_r differs from theoretical because of posttranslationally added glycans; # fragments F_1 and F_2 are produced from F_0 by cleavage with cell protease furin which recognizes RRHKR motif

Table S2. Proteins coded by MUV genome (L-Zagreb strain, GenBank: AY685921.1)

protein	CDS	#aa	M_r	*p*I	GI (NCBI)	UniProt entry
nucleoprotein, NP	146 – 1795	549	61336	5.24	55775564	Q5SC49
phosphoprotein, P	1979 – 3152	391	41595	7.82	55775566	Q5SC57
V	1979 – 2653	224	24096	8.86	55775564	Q5SC47
I	1979 – 2490	171	18302	10.04	55775568	Q5SC46
matrix protein, M	3228 – 4481	375	41580	9.52	55775568	Q5SC54
fusion protein, F_0*	4546 – 6162	538	58730	8.13	55775569	Q5SC53
fusion protein fragment 1, $F_1^{\#}$	-	436	47345	5.99	-	-
fusion protein fragment 2, $F_2^{\#}$	-	102	11402	9.67	-	-
small hydrophobic protein, SH	6268 – 6441	57	6833	9.82	55775560	Q5SC52
hemagglutinin-neuraminidase, HN*	6614 – 8362	582	64127	7.79	55775571	Q5SC51
large polymerase, L	8468 – 11815	2261	256819	6.18	55775572	Q5SC50

CDS - coding sequence; aa – amino acid; * - glycoproteins, real M_r differs from theoretical because of posttranslationally added glycans; $^{\#}$ fragments F_1 and F_2 are produced from F_0 by cleavage with cell protease furin which recognizes RRHKR motif

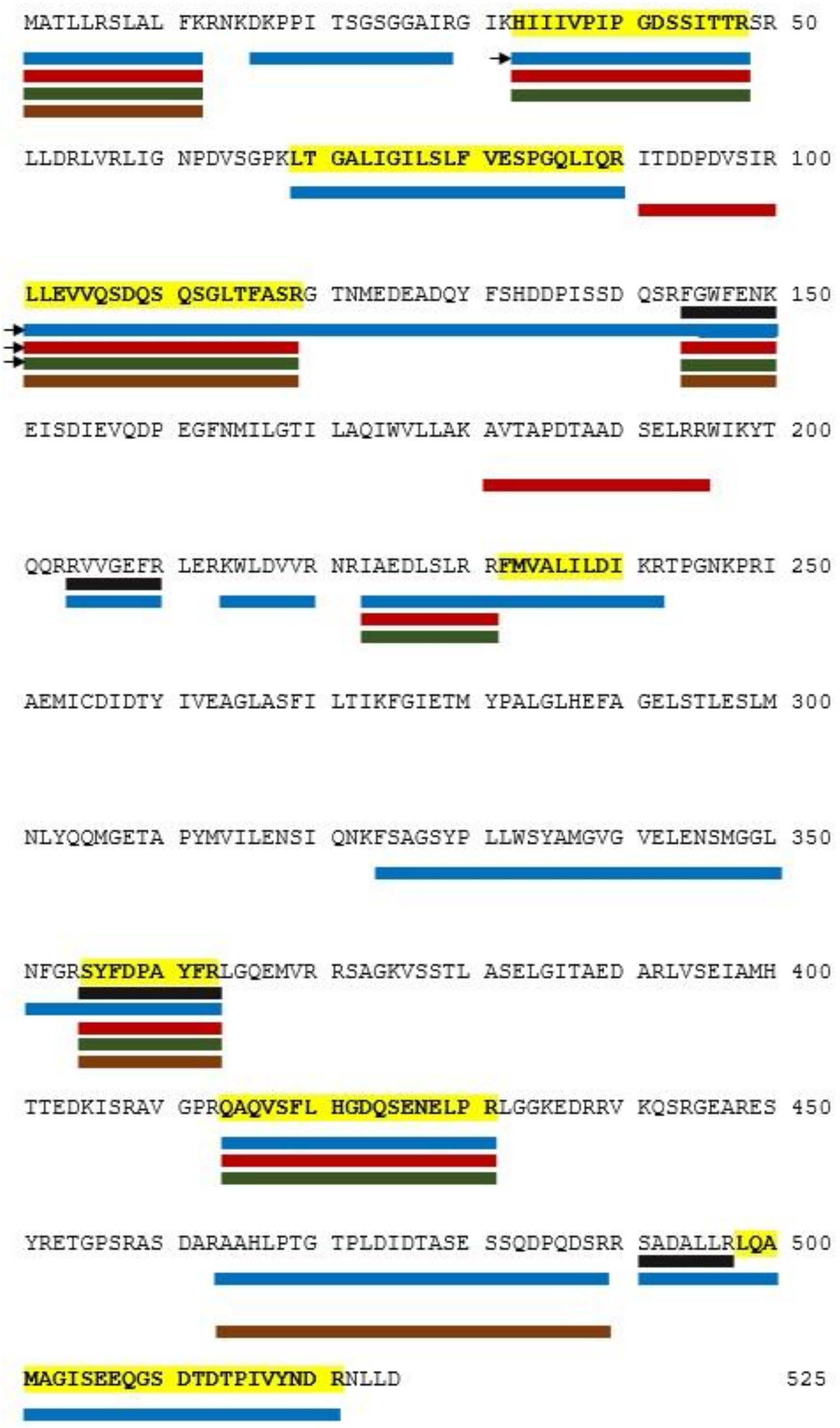

Figure S1. Sequence coverage of MEV N bands from UC purified MEV (Fig. 1(c)). Black line – apparent molecular mass 59 kDa, blue line – apparent molecular mass 56 kDa, red line – apparent molecular mass 53 kDa, green line – apparent molecular mass 51 kDa, brown line – apparent molecular mass 42 kDa. Peptides for which the signals were observed in the PMF spectra of a protein form are underlined in corresponding color. Peptides sequenced by MS/MS are highlighted yellow in the sequence. Arrows denote in which protein forms that peptide was sequenced, if not in all.

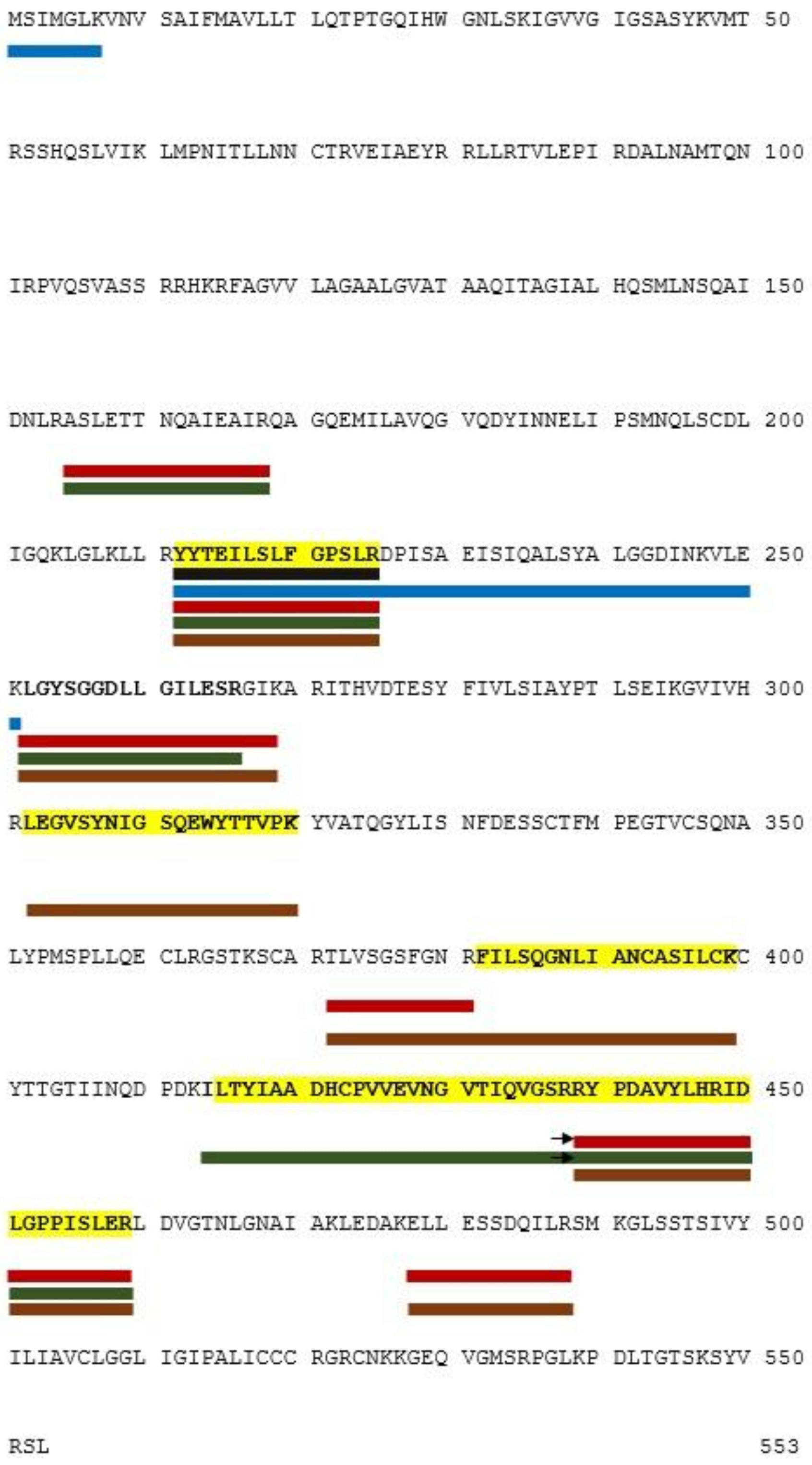

Figure S2. Sequence coverage of MEV F bands from UC purified MEV (Fig. 1(c)). Black line – apparent molecular mass 59 kDa, blue line – apparent molecular mass 56 kDa, red line – apparent molecular mass 53 kDa, green line – apparent molecular mass 51 kDa, brown line – apparent molecular mass 42 kDa. Peptides for which the signals were observed in the PMF spectra of a protein form are underlined in corresponding color. Peptides sequenced by MS/MS are highlighted yellow in the sequence. Arrows denote in which protein forms that peptide was sequenced, if not in all.

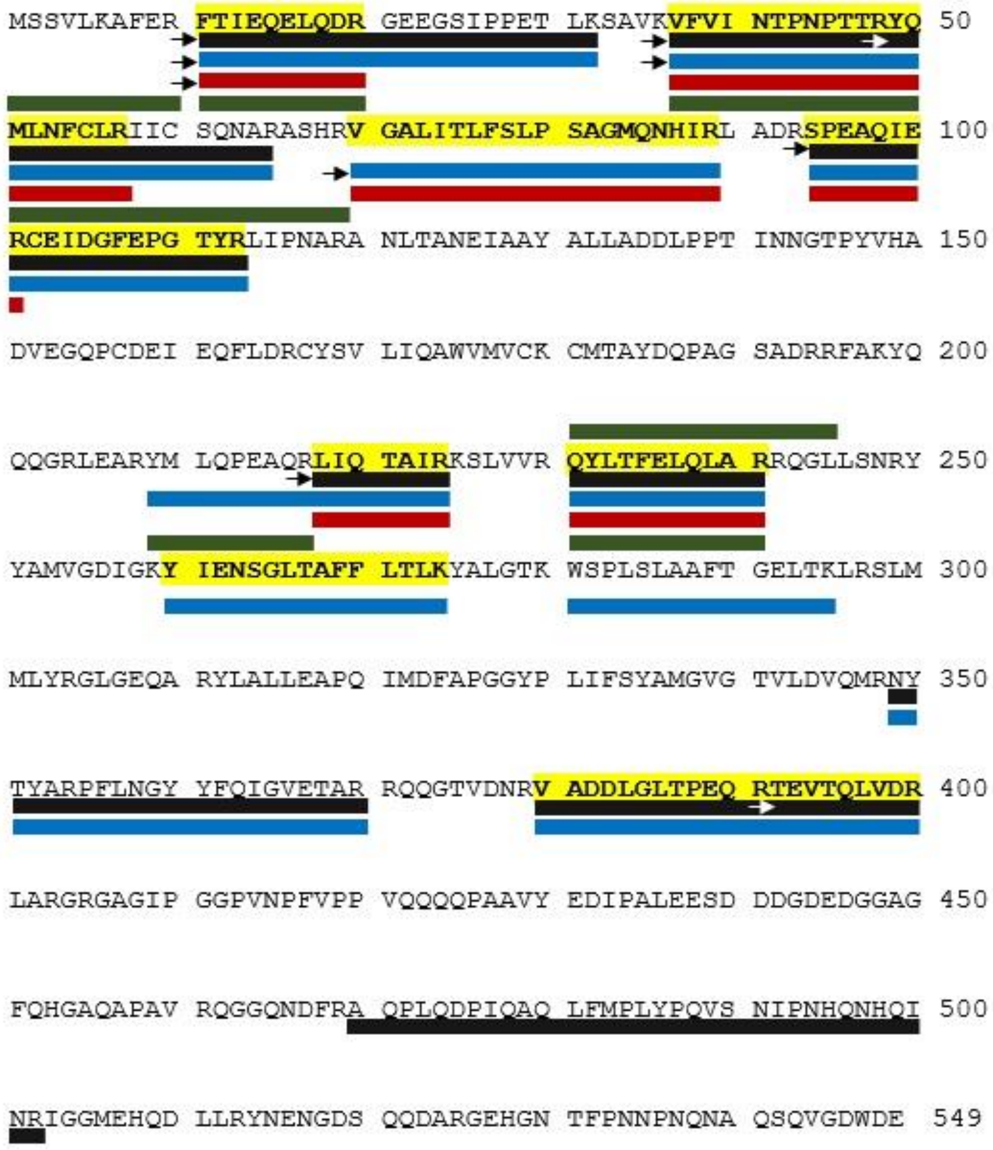

Figure S3. Sequence coverage of MUV NP bands from UC purified MUV (Fig. 2(c)). Black line – N apparent molecular mass 61 kDa, blue line - N apparent molecular mass 55 kDa, red line - N apparent molecular mass 53 kDa, green line - N apparent molecular mass 39 kDa. Peptides for which the signals were observed in the PMF spectra of a protein form are underlined in corresponding color. Peptides sequenced by MS/MS are highlighted yellow in the sequence. Arrows denote in which protein forms that peptide was sequenced, if not in all.

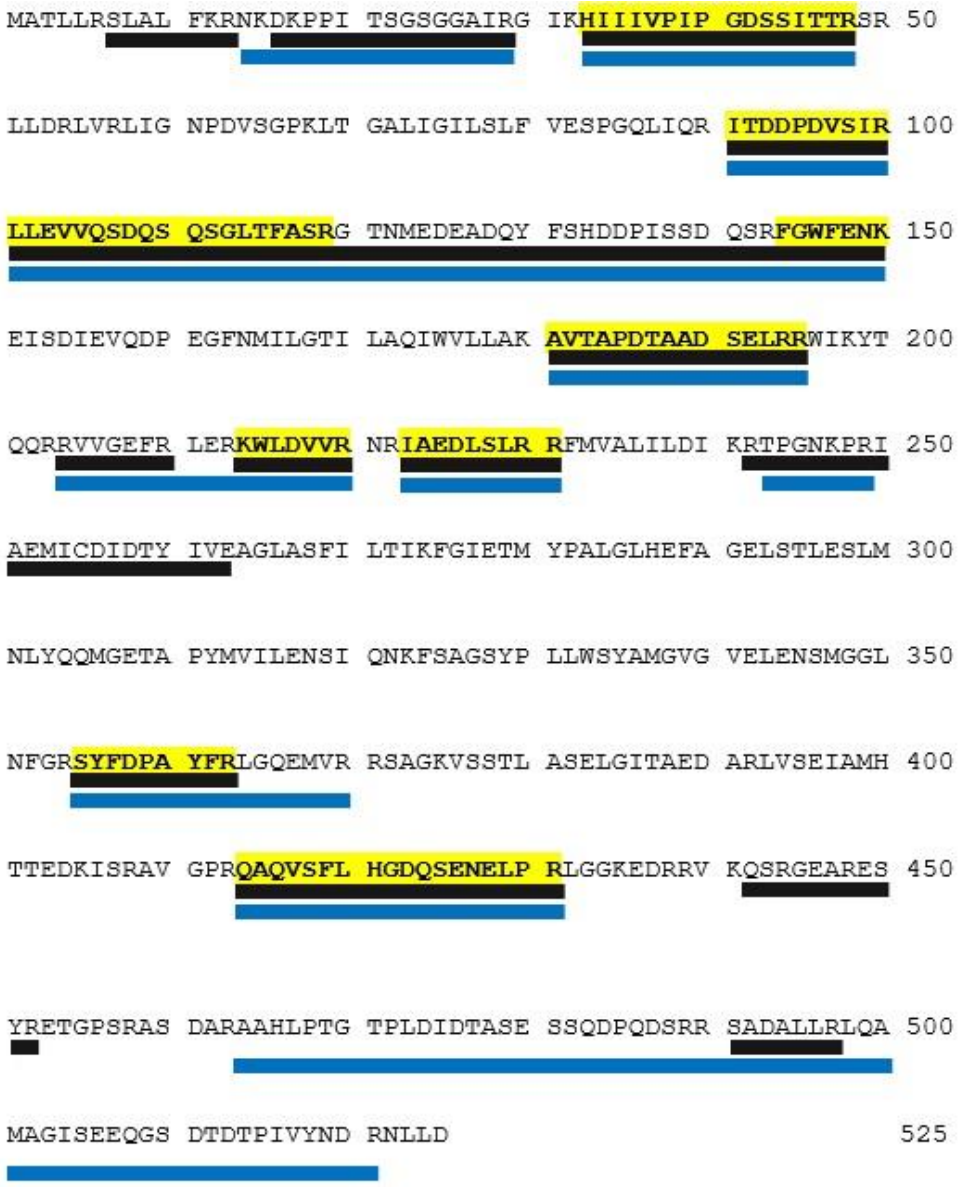

Figure S4. Sequence coverage of MEV N bands from E2 fraction of HIC purified MEV (Fig. 3). Black line – apparent molecular mass 59 kDa, blue line – apparent molecular mass 55 kDa. Peptides for which the signals were observed in the PMF spectra of a protein form are underlined in corresponding color. Peptides sequenced by MS/MS are highlighted yellow in the sequence.

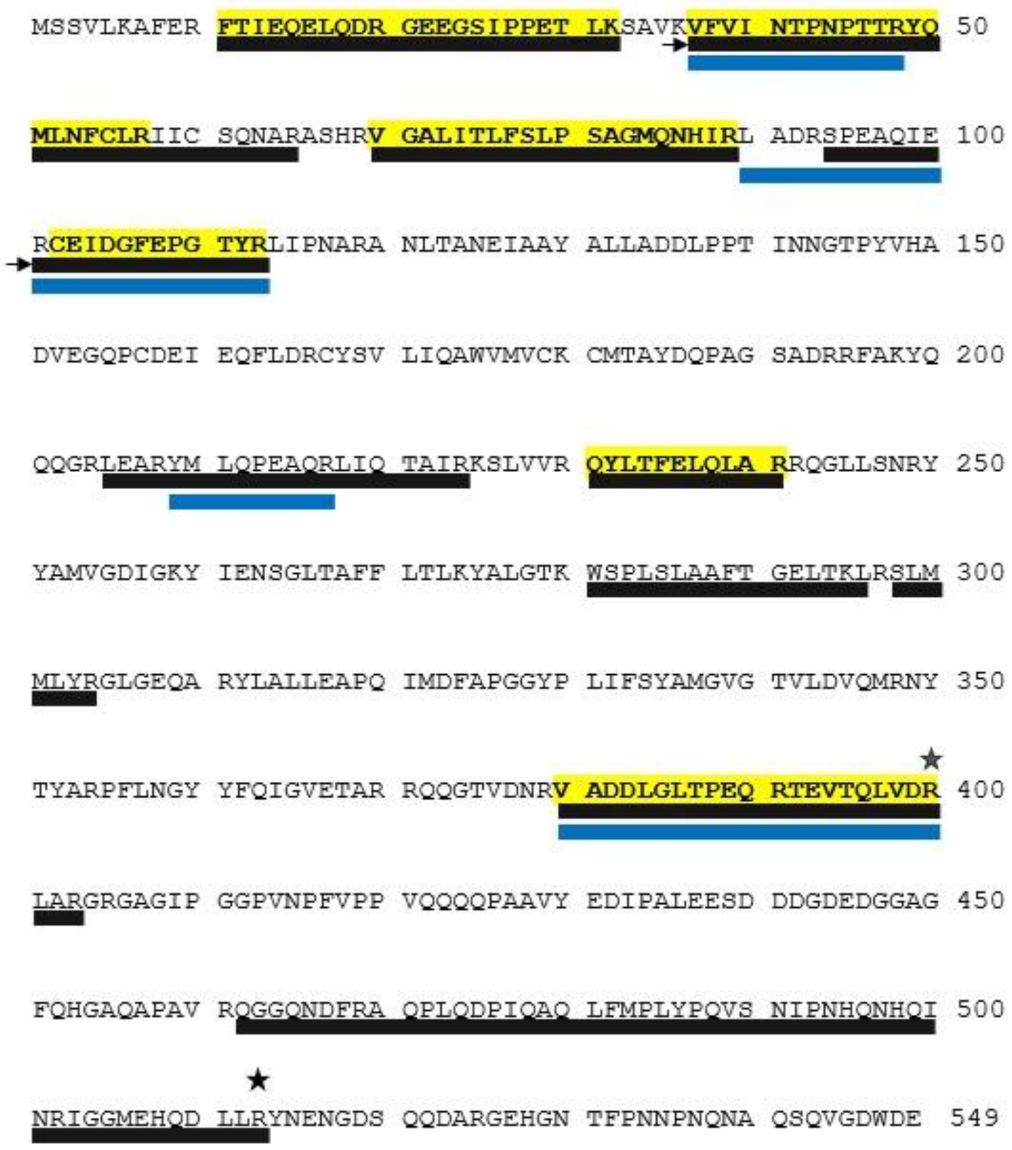

Figure S5. Sequence coverage of MUV NP bands from fraction E2 of HIC purified MUV (Fig. 4). Black line – apparent molecular mass 56 kDa, blue line – apparent molecular mass 46 kDa. Peptides for which the signals were observed in the PMF spectra of a protein form are underlined in corresponding color. Peptides sequenced by MS/MS are highlighted yellow in the sequence. Arrows denote in which protein forms that peptide was sequenced, if not in all. Grey star marks position 400 at which C-truncation yields a 45 kDa protein, whereas black star denotes position 513 at which C-truncation yields a 57 kDa protein.

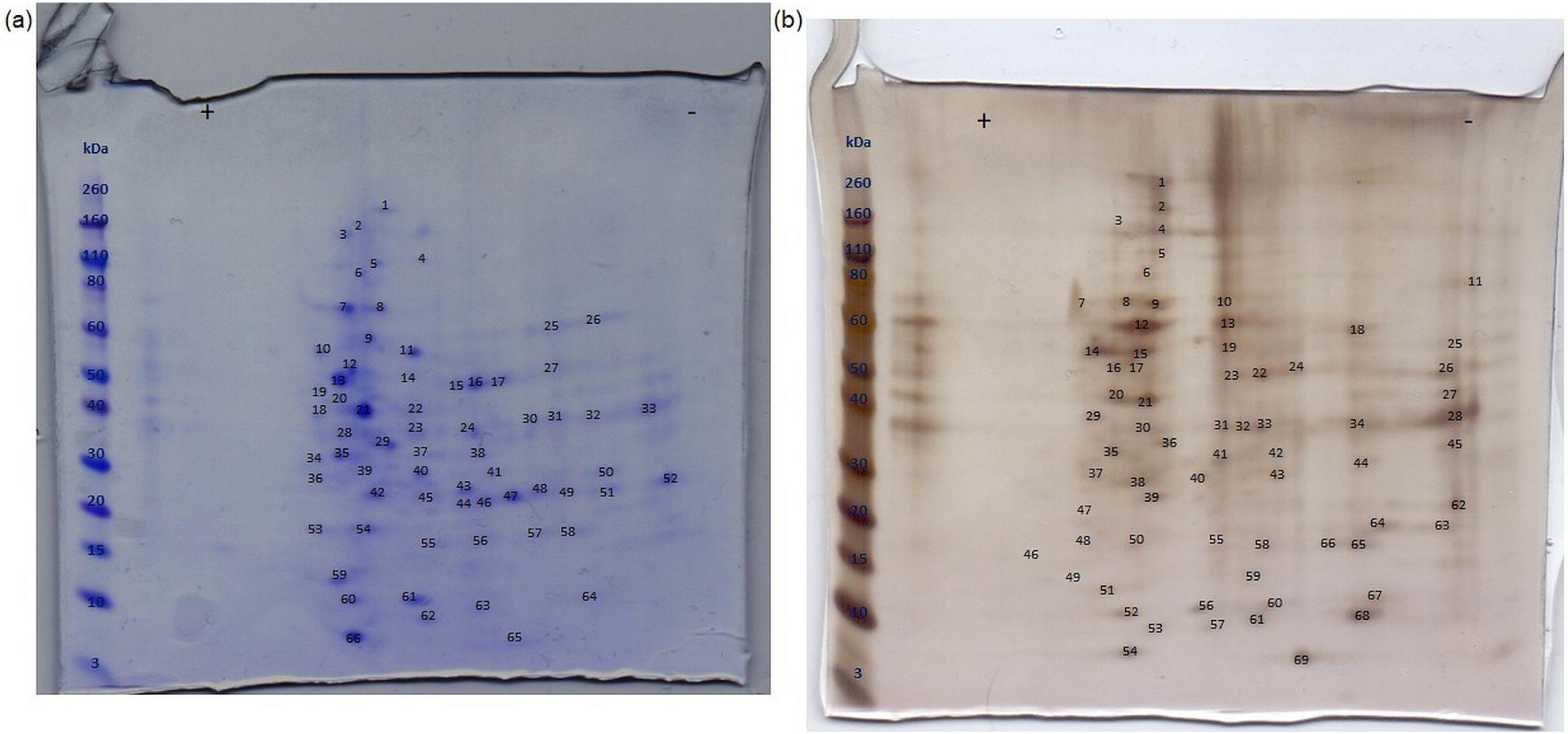

Figure S6. 2D gel electrophoresis of (a) MUV (same sample as in Fig. 2(c)) and (b) MEV (same sample as in Fig. 1(c)) purified by UC with protein annotations after MALDI-TOF/TOF MS analysis. List of identified proteins is given in Table S1.

Table S3. List of proteins detected in MEV and MUV samples purified by UC and analysed by 2D gel electrophoresis

MUV		MEV	
Spot number	protein	Spot number	protein
1	plexin B2	1	ni
2	ni	2	ni
3	integrin αV	3	ni
4	integrin α3	4	ni
5	non-muscle myosin heavy chain	5	ni
6	ni	6	N
7	HSPA5	7	ni
8	HSP70 - mortalin	8	HSPA5
9	HSP60, NP	9	HSP71
10	PDIA3	10	moesin
11	PDIA3	11	ni
12	tubulin α	12	P
13	tubulin β	13	P, N
14	heterogeneous nuclear ribonucleoprotein	14	N, P
15	α-enolase, P	15	N, P
16	α-enolase	16	tubulin α
17	α-enolase	17	ni
18	vimentin, actin	18	ni
19	tubulin β	19	N
20	actin, HSP71	20	actin
21	actin	21	actin
22	actin	22	ni
23	annexin A2, α-enolase	23	ni
24	annexin A2, aldolase reductase	24	α-enolase
25	NP	25	ni
26	NP	26	ni
27	ni	27	actin
28	annexin A2	28	M, P
29	annexin A4	29	P, M
30	annexin A2	30	ni
31	annexin A2	31	M
32	annexin A2	32	ni
33	galectin-3	33	ni
34	ni	34	ni
35	annexin A5	35	annexin A5
36	14-3-3 gamma protein	36	annexin A4
37	ni	37	14-3-3 gamma protein
38	annexin A2	38	ni
39	chloride intracellular channel protein	39	ni

Table S3. Continued

MUV<br>Spot number	MEV<br>protein	MUV<br>Spot number	MEV<br>protein
40	HSPβ1	40	HSPβ1
41	triosephosphate isomerase, phosphoglycerate mutase	41	P/V
42	ubiquitin carboxy-terminal hydrolase, sorcin	42	ni
43	voltage dependant anion selective channel protein (porin 31)	43	triosephosphate isomerase
44	superoxide dismutase	44	HSP71
45	GST, annexin A1	45	moesin
46	peroxiredoxin, superoxide dismutase	46	ni
47	superoxide dismutase	47	ni
48	P, superoxide dismutase	48	myosin regulatory protein, light chain
49	ni	49	ni
50	P	50	ni
51	superoxide dismutase, peroxiredoxin	51	galectin-1
52	peroxiredoxin	52	ni
53	vimentin	53	ni
54	annexin A2, Rab7 GTPase	54	ni
55	ni	55	ni
56	CypA	56	ni
57	CypA	57	ni
58	CypA	58	CypA
59	galectin-1	59	ni
60	ni	60	ni
61	protein S100-A11	61	ni
62	protein S100-A11	62	peroxiredoxin
63	protein S100-A11	63	cofilin
64	ni	64	cofilin
65	ni	65	CypA
66	ni	66	CypA
		67	ni
		68	ni
		69	ribosomal protein S27a

ni – not identified
